# Methotrexate Provokes Disparate Folate Metabolism Gene Expression and Alternative Splicing in Ex Vivo Monocytes and GM-CSF- and M-CSF-Polarized Macrophages

**DOI:** 10.3390/ijms24119641

**Published:** 2023-06-01

**Authors:** Ittai B. Muller, Marry Lin, Robert de Jonge, Nico Will, Baltasar López-Navarro, Conny van der Laken, Eduard A. Struys, Cees B. M. Oudejans, Yehuda G. Assaraf, Jacqueline Cloos, Amaya Puig-Kröger, Gerrit Jansen

**Affiliations:** 1 Department of Laboratory Medicine, Amsterdam University Medical Center, 1105 AZ Amsterdam, The Netherlands; i.muller@amsterdamumc.nl (I.B.M.); m.lin@amsterdamumc.nl (M.L.); r.dejonge1@amsterdamumc.nl (R.d.J.); e.struys@amsterdamumc.nl (E.A.S.); cbm.oudejans@gmail.com (C.B.M.O.); 2 Facility for Environment and Natural Science, Brandenburg Technical University Cottbus-Senftenberg, 01968 Senftenberg, Germany; nicowill22111@gmail.com; 3 Laboratorio de Inmuno-Metabolismo e Inflamación, Instituto de Investigación Sanitaria Gregorio Marañón, Hospital Gregorio Marañón, 28007 Madrid, Spain; baltasar.lopez@iisgm.com (B.L.-N.); amaya.puig@iisgm.com (A.P.-K.); 4 Department of Rheumatology and Clinical Immunology, Amsterdam Rheumatology and Immunology Center, Amsterdam University Medical Center–location VUmc, 1081 HV Amsterdam, The Netherlands; j.vanderlaken@amsterdamumc.nl; 5 The Fred Wyszkowski Cancer Research Laboratory, Department of Biology, Technion-Israel Institute of Technology, Haifa 3200003, Israel; assaraf@technion.ac.il; 6 Department of Hematology, Amsterdam University Medical Center–location VUmc, Cancer Center Amsterdam, 1081 HV Amsterdam, The Netherlands; j.cloos@amsterdamumc.nl

**Keywords:** methotrexate, macrophages, gene expression, alternative splicing, folate metabolism, folylpolyglutamate synthetase

## Abstract

Macrophages constitute important immune cell targets of the antifolate methotrexate (MTX) in autoimmune diseases, including rheumatoid arthritis. Regulation of folate/MTX metabolism remains poorly understood upon pro-inflammatory (M1-type/GM-CSF-polarized) and anti-inflammatory (M2-type/M-CSF-polarized) macrophages. MTX activity strictly relies on the folylpolyglutamate synthetase (FPGS) dependent intracellular conversion and hence retention to MTX-polyglutamate (MTX-PG) forms. Here, we determined FPGS pre-mRNA splicing, FPGS enzyme activity and MTX-polyglutamylation in human monocyte-derived M1- and M2-macrophages exposed to 50 nmol/L MTX ex vivo. Moreover, RNA-sequencing analysis was used to investigate global splicing profiles and differential gene expression in monocytic and MTX-exposed macrophages. Monocytes displayed six–eight-fold higher ratios of alternatively-spliced/wild type FPGS transcripts than M1- and M2-macrophages. These ratios were inversely associated with a six–ten-fold increase in FPGS activity in M1- and M2-macrophages versus monocytes. Total MTX-PG accumulation was four-fold higher in M1- versus M2-macrophages. Differential splicing after MTX-exposure was particularly apparent in M2-macrophages for histone methylation/modification genes. MTX predominantly induced differential gene expression in M1-macrophages, involving folate metabolic pathway genes, signaling pathways, chemokines/cytokines and energy metabolism. Collectively, macrophage polarization-related differences in folate/MTX metabolism and downstream pathways at the level of pre-mRNA splicing and gene expression may account for variable accumulation of MTX-PGs, hence possibly impacting MTX treatment efficacy.

## 1. Introduction

In the past 75 years, the folate antagonist methotrexate (MTX) has acquired a prominent position in the treatment of malignant as well as non-malignant disorders such as rheumatoid arthritis (RA) and juvenile idiopathic arthritis (JIA) [1,2]. Based upon mechanisms which remain incompletely understood, the anti-arthritic activity of MTX is thought to be exerted via inhibition of key enzymes in folate and purine metabolism, which ultimately leads to suppression of cytokine-driven inflammatory signaling pathways in various (activated) immune cells [3,4,5,6,7,8,9]. A critical determinant in the activity of MTX is the folylpolyglutamate synthetase (FPGS)-catalyzed conversion to its polyanionic MTX-polyglutamate (MTX-PG) forms. This promotes intracellular retention and results in enhanced potent inhibition of target enzymes as compared to non-polyglutamylated MTX [10,11]. In fact, the crucial role of FPGS has been demonstrated via diminished or loss of FPGS activity, which was found to result in the lack of MTX responsiveness and acquired MTX resistance in both RA and cancer [12,13,14,15]. 

It has been reported that the catalytic activity of FPGS may vary several orders of magnitude among distinct cells; it may be low in resting/non-proliferating (immune) cells, but high in rapidly proliferating tumor cells (leukemias and solid tumors) [12,16,17]. Recent studies indicated that aberrant pre-mRNA splicing of FPGS is a mechanism underlying loss of FPGS activity due to the formation of splice variants undergoing premature translation termination [18,19,20]. Multiple FPGS splice variants were identified in leukemia cells and peripheral blood mononuclear cells (PBMCs) of RA patients. One of these splice variants, a partial retention of intron 8 (8PR), was associated with diminished FPGS activity and reduced therapy response to MTX [20,21,22]. 

Considering the emerging role of aberrant pre-mRNA splicing in drug resistance [20,23], we investigated FPGS splicing (8PR/WT) in macrophages, one of the main representative immune cell types known to be targeted by MTX therapy in RA [6,7,24,25,26,27]. First, FPGS (8PR/WT) splicing was examined during ex vivo monocyte to macrophage differentiation and polarization to pro-inflammatory M1-type (GM-CSF-primed) and anti-inflammatory M2 type (M-CSF-primed) macrophages [28,29,30,31]. Second, the impact of FPGS splicing for FPGS activity and MTX-PG formation was determined in M1- and M2-type macrophages following exposure to low-dose MTX. Lastly, complementary RNA sequencing (RNA-seq) was performed to monitor global MTX-induced alterations in general splicing and differential gene expression profiles in monocyte-derived M1- and M2-type macrophages. The aim of these studies was to gain a broader insight into how MTX impacts the processes of monocyte to macrophage differentiation and polarization that could play a role in the therapeutic effect of MTX in RA treatment. 

## 2. Results

### 2.1. FPGS Pre-mRNA Splicing, FPGS Activity and MTX-Polyglutamylation in Monocytes and M1- and M2-Type Macrophages

**Figure 1:**
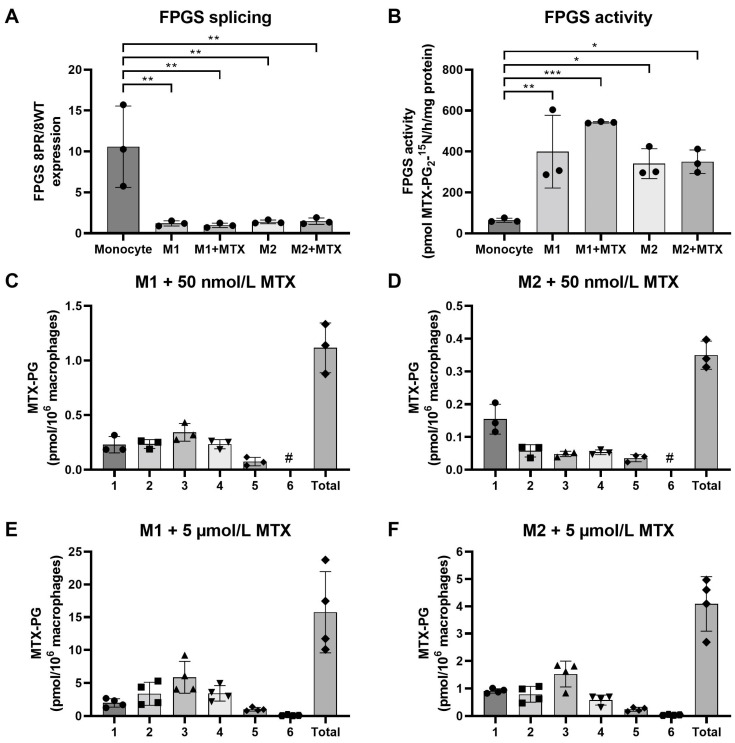
(**A**) Expression levels of FPGS 8PR/WT in monocytes (Mo), M1–MØ (7 day, ±50 nmol/L MTX) and M2–MØ (7 day, ±50 nmol/L MTX). (**B**) FPGS catalytic activity in monocytes, M1–MØ (7 day, ±50 nmol/L MTX) and M2–MØ (7 day, ± 50 nmol/L MTX). Formation and accumulation of MTX-PG after 7 days exposure to 50 nmol/L MTX in (**C**) M1–MØ and (**D**) M2–MØ, and after 7 days exposure to 5 µmol/L MTX in (**E**) M1–MØ and (**F**) M2–MØ. Results are expressed as a mean ± SD for 3–4 individual donors. * *p* < 0.05, ** *p* < 0.01, *** *p* < 0.001. #: not detectable.

To explore whether FPGS splicing is a determinant in the regulation of FPGS activity in monocytes and polarized macrophages, ratios of FPGS 8PR/WT were determined in monocytes, GM-CSF (M1-type) macrophages (MØ) and M-CSF (M2-type) MØ with or without 7 days of exposure to low-dose (50 nmol/L) MTX (Figure 1A). A markedly high 8PR/WT ratio is observed in monocytes, which decreases significantly (six- to eight-fold) following differentiation and polarization to M1- and M2-type MØ. Exposure to low-dose MTX had no additional impact. 

Anticipating that a higher ratio of FPGS 8PR/8WT might translate into impaired FPGS activity [20,21,22], we tested this by analysis of FPGS enzyme activity (Figure 1B). Indeed, an inverse pattern of 8PR/WT and FPGS activity was observed, with the lowest FPGS activity in monocytes, which was increased six- to ten-fold in M1- and M2-type MØ. FPGS activity was moderately increased in M1–MØ (+MTX) compared to M1–MØ (-MTX); however, this difference did not reach statistical significance. MTX exposure did not alter FPGS activity in M2–MØ. Conceivably, the increase in FPGS activity during monocyte macrophage differentiation/polarization reflects the need for folate cofactors for biosynthetic processes [10,17]. 

Next, we determined whether FPGS activity is correlated with the formation and accumulation of MTX-PGs in M1–MØ and M2–MØ following 7 days of exposure to 50 nmol/L MTX (Figure 1C). M1–MØ cells revealed the formation and accumulation of MTX-PG_2_, MTX-PG_3_ (the dominant form) and MTX-PG_4_. In contrast, M2–MØ hardly accumulated any MTX-PGs; most intracellular MTX is MTX-PG_1_ and a small fraction of MTX-PG_2_ (Figure 1D). Since FPGS activities in M1- and M2–MØ are comparable (Figure 1B), the markedly diminished accumulation of MTX-PGs in M2–MØ at low-dose MTX may be due to impaired MTX uptake as compared to M1–MØ. To this end, we also examined whether this apparent impaired transport may be overcome after exposure to a high dose (i.e., 5 μmol/L) MTX. For M1–MØ, total MTX-PG accumulation was 14-fold higher after high-dose vs. low-dose MTX exposure with a similar MTX-PG distribution profile (Figure 1E). Total MTX-PG accumulation in M2–MØ was also markedly increased after high-dose MTX (12-fold over low-dose MTX) along with a MTX-PG distribution profile (MTX-PG_3_ dominant form) now being similar to the M1–MØ (Figure 1F). Together, these results indicate that FPGS splicing is a contributing factor in regulating FPGS activity during monocyte to macrophage polarization, and that FPGS activity and differential MTX uptake profiles underlie variable MTX-PG accumulation in M1–MØ and M2–MØ. 

### 2.2. Differential Splicing Profiles in Monocytes, M1–MØ and M2–MØ

**Figure 2:**
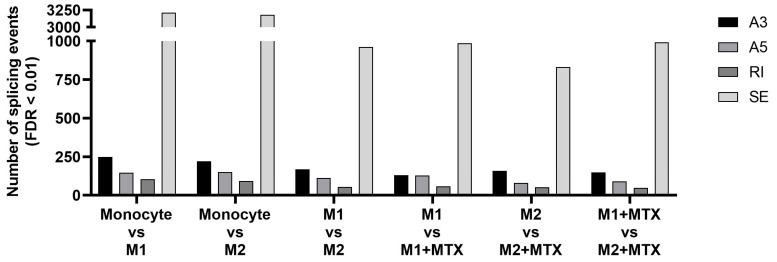
Number of differential expression (FDR < 0.01) of 4 splicing types: Skipped Exon (SE), Retained Intron (RI), Alternative 3′ Splice Site (A3) and Alternative 5′ Splice Site (A5) for: Mo vs. M1–MØ, Mo vs. M2–MØ, M1–MØ vs. M2–MØ, M1–MØ vs. M1–MØ + MTX, M2–MØ vs. M2–MØ + MTX and M1–MØ + MTX vs. M2–MØ + MTX. Results depicted are the means of 3 individual donors.

To determine whether MTX treatment induces specific splicing patterns, RNA-seq analysis was performed to determine global differential splicing profiles in M1–MØ vs. M1–MØ + MTX as well as M2–MØ vs. M2–MØ + MTX. Figure 2 depicts the number of significant splicing events (FDR < 0.01) per category for monocytes vs. polarized MØ +/− MTX. The majority of splice events involved SE, followed by A3SS, A5SS and RI. The highest total number of differential splice events were observed for monocytes vs. M1–MØ (3210 events) and monocytes vs. M2–MØ (3179 events) and three-fold lower numbers for M1–MØ vs. M2–MØ (961 events), M1–MØ vs. M1–MØ + MTX (986 events), M2–MØ vs. M2–MØ + MTX (832 events), and M1–MØ + MTX vs. M2–MØ + MTX (991 events).

**Figure 3:**
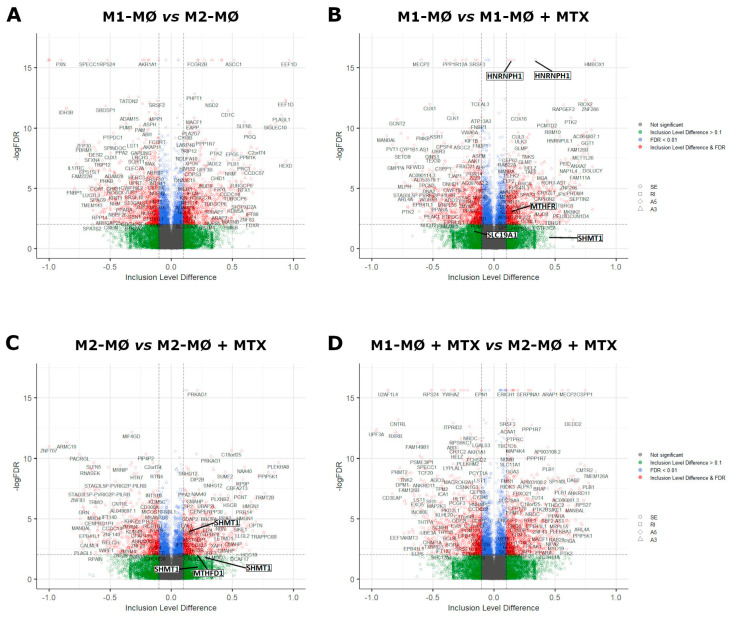

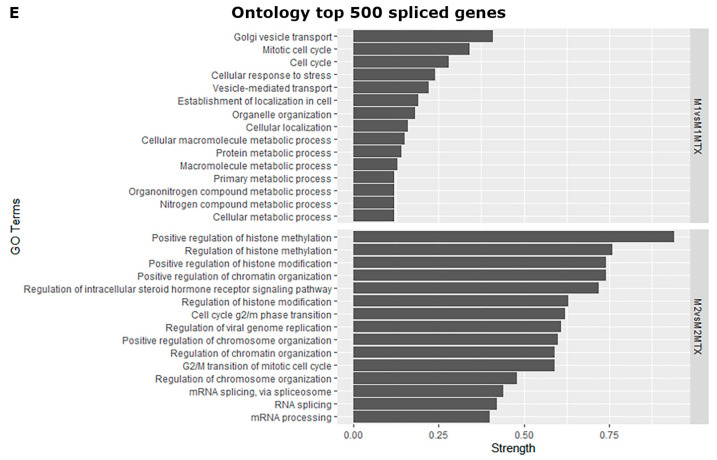
Volcano plots of differentially spliced events (SE, RI, A3 and A5) for (**A**) M1–MØ vs. M2–MØ (total detected splice variants: 34,426), (**B**) M1–MØ vs. M1–MØ + MTX (total detected splice variants: 35,800), (**C**) M2–MØ vs. M2–MØ + MTX (total detected splice variants: 33,990), and (**D**) M1–MØ + MTX vs. M2–MØ + MTX (total detected splice variants: 35,558). Results depicted are the means of 3 individual donors. (**E**) Gene ontology terms top 500 differentially spliced genes of comparisons B and C. Strength scores show markedly stronger involvement of top differentially spliced genes in M2–MØ vs. M2–MØ + MTX (>0.7) than in M1–MØ vs. M1–MØ + MTX (<0.4) indicating higher confidence of potential protein interactions in this condition.

Profiles of differentially spliced genes of the comparisons shown in Figure 2 are presented in Figure 3. Volcano plots for the monocyte vs. M1–MØ and monocyte vs. M2–MØ comparisons are shown in Supplemental Figure S1. The top 100 most differentially spliced genes (based on Inclusion Level Difference > 0.1) are annotated in Supplementary Tables S1–S6. In the context of this study, we checked whether additional folate-related genes were among the top 100 of spliced genes. Splicing of methylene tetrahydrofolate reductase (MTHFR) and the reduced folate carrier (RFC, SLC19A1) were annotated in M1–MØ vs. M1–MØ + MTX (Figure 3B/Supplemental Table S4). In addition, serine hydroxymethyltransferase 1 (SHMT1) and methylenetetrahydrofolate dehydrogenase 1 (MTHFD1) were annotated in M2–MØ vs. M2–MØ + MTX (Figure 3C/Supplemental Table S5). Other selected spliced genes included those involved in the splicing process itself, such as heterogeneous nuclear ribonucleoprotein H1 (HNRNP1) and splicing factor 3b subunit 1 (SF3B1) (Figure 3B/Supplemental Table S4). FPGS splicing was annotated in monocytes vs. M1–MØ and M2–MØ (Supplemental Figure S1). In concordance with Figure 1A, differential splicing analysis showed a significant splice event (FPGS 8PR, annotated as an alternative 5’ splice site) of the FPGS RNA transcript in the monocytes vs. M1–MØ (FDR = 2.2 × 10^−8^, Inclusion Level Difference = 0.193) and the monocyte vs. M2–MØ comparison (FDR = 8.5 × 10^−9^ and Inclusion Level Difference = 0.193). No significant splice event was seen in the other comparisons, similar to Figure 1A. Additionally, top differentially spliced genes for M1–MØ vs. M1–MØ + MTX and M2–MØ vs. M2–MØ + MTX were analyzed by String, revealing predominantly genes involved in Golgi vesicle transport and cell cycle for M1–MØ vs. M1–MØ + MTX as well as histone methylation/modification for M2–MØ vs. M2–MØ + MTX (Figure 3E). 

### 2.3. Differential Gene Expression (DGE) Profiling of Monocytes, M1–MØ and M2–MØ (with and without MTX Exposure)

**Figure 4:**
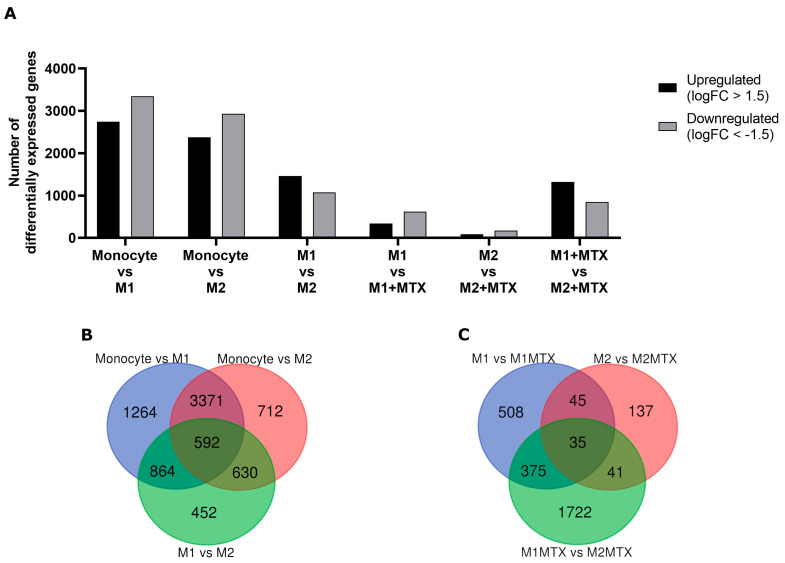
(**A**) The number of significant up- and downregulated (FDR < 0.05, logFC > 1.5) genes for Mo vs. M1–MØ, Mo vs. M2–MØ, M1–MØ vs. M2–MØ, M1–MØ vs. M1–MØ + MTX, M2–MØ vs. M2–MØ + MTX, M1–MØ + MTX vs. M2–MØ + MTX, and M1–MØ + MTX vs. M2–MØ + MTX. Results depicted are the means of 3 individual donors. (**B**) Venn diagram comparing differentially expressed genes for Mo vs. M1–MØ, Mo vs. M2–MØ, M1–MØ vs. M2–MØ. (**C**) Venn diagram comparing differentially expressed genes for M1–MØ vs. M1–MØ + MTX, M2–MØ vs. M2–MØ + MTX, M1–MØ + MTX vs. M2–MØ + MTX, M1–MØ + MTX vs. M2–MØ + MTX.

We next analyzed RNA-seq data for gene expression profiles in monocytes, M1–MØ and M2–MØ in the presence or absence of MTX. Figure 4A shows the numbers of up- and downregulated genes for six groups of cells and MTX exposure conditions. The highest numbers of differentially expressed genes (>2300) were observed in monocytes vs. M1–MØ and monocytes vs. M2–MØ; whereas the lowest numbers (88–170) were found for M2–MØ vs. M2–MØ + MTX. Comparisons of differentially expressed genes under conditions with and without MTX exposure revealed a modest overlap of genes impacted by MTX in M1–MØ vs. M2–MØ (Figure 4B,C). 

**Figure 5:**
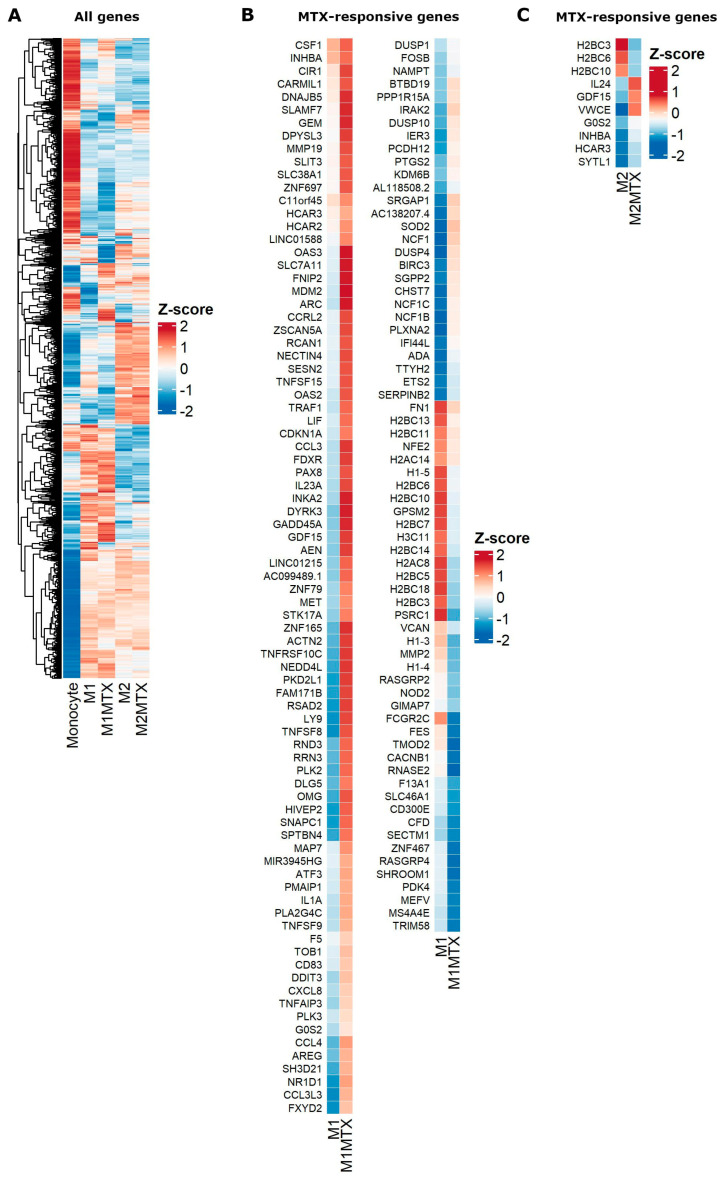
Heat maps for (**A**) global differential gene expression for Mo, M1–MØ, M1–MØ + MTX, M2–MØ and M2–MØ + MTX. (**B**) Significant MTX-responsive genes for M1–MØ and M1–MØ + MTX (FDR < 0.05, logFC > 1.5, average expression > 1.0). (**C**) Significant MTX-responsive genes for M2–MØ and M2–MØ + MTX (FDR < 0.05, logFC > 1.5, average expression > 1.0). Results depicted are the means of 3 individual donors.

A heat map overview of DGE in monocytes, M1–MØ and M2–MØ in the absence or presence of MTX exposure is shown in Figure 5A and ranking of the top 100 up- and downregulated genes is depicted in Supplementary Table S7. Distinct patterns of DGE are noted for monocytes vs. M1–MØ and M2–MØ, with more overlap between M1–MØ and M2–MØ, and selected differences for MTX-exposed M1–MØ and M2–MØ. To further identify MTX-responsive genes in M1–MØ and M2–MØ, genes were selected based upon an FDR < 0.05, logFC > 1.5 and average gene expression > 1.0, revealing significant transcriptional differences for 152 genes in M1–MØ (Figure 5B) and for eight genes in M2–MØ (Figure 5C). 

**Figure 6:**
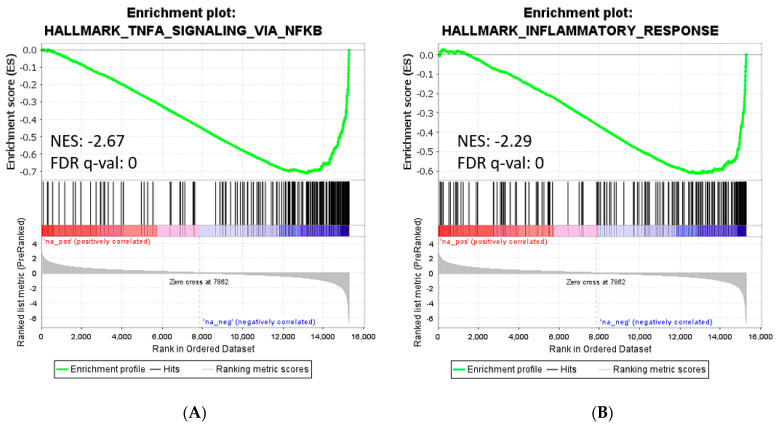
Gene set enrichment analysis on the ranked comparison of M1–MØ vs. M1–MØ + MTX. Enrichment plot for significant signature: TNFα Signaling via NFκB (**A**) and Inflammatory Response (**B**). Normalized enrichment scores (NES) indicate the proportionality of genes included in the signature.

GSEA of the pre-ranked comparison of the transcriptome of M1–MØ vs. M1–MØ + MTX revealed 41 gene sets being significantly enriched in M1–MØ + MTX (FDR q-val < 0.05) (Supplemental Table S8). The top 10 gene sets are shown in this table, among which several are related to immune response, suggest that M1–MØ + MTX acquired an enhanced pro-inflammatory phenotype. Two relevant enrichment plots: “TNFA_SIGNALING_VIA_NFKB” and “INFLAMMATORY_RESPONSE” are shown in Figure 6.

**Figure 7:**
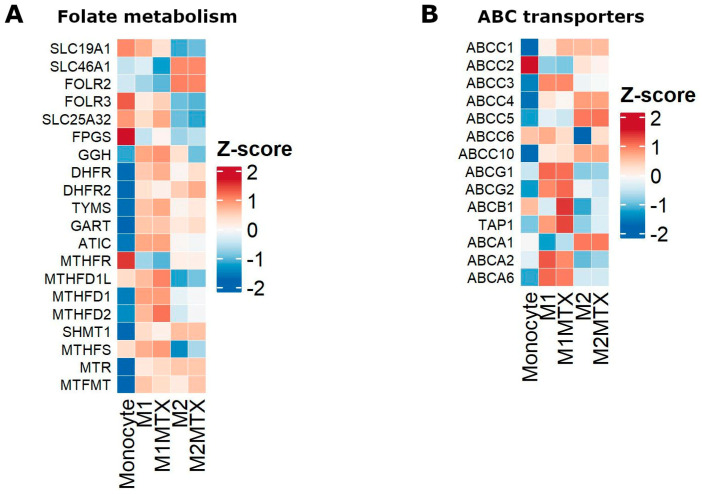
Heat maps of differential gene expression of Mo, M1–MØ, M1–MØ + MTX, M2–MØ and M2–MØ + MTX for (**A**) genes encoding for transporters and enzymes in cytosolic and mitochondrial folate metabolism, and (**B**) genes encoding for drug efflux transporters of the ABC transporter family. Results depicted are the means of 3 individual donors.

Gene expression data were also used to compile relevant genes in cellular pharmacology of MTX, i.e., folate metabolism genes (Figure 7A) and drug efflux transporters of the ABC transporter family (Figure 7B) of which ABCC1-5 and ABCG2 are involved in folate/MTX drug efflux [26,32,33,34,35,36]. The DGE profile for folate metabolism genes shows a clear distinction between M1–MØ and M2–MØ with generally higher expression levels of cytosolic (DHFR, TS, ATIC) and mitochondrial folate enzymes (MTHFD2) in M1–MØ over M2–MØ. Likewise, expression of the plasma membrane folate/MTX transporter RFC (SLC19A1) and mitochondrial folate transporter (MFT/SCL25A32) is increased in M1–MØ over M2–MØ. Two other folate carriers, FOLR2/FRβ and PCFT (SLC46A1), are increasingly expressed in M2–MØ over M1–MØ. Low-dose MTX exposure confers a modest increase in the expression of MFT (SLC25A32) and enzymes (MTHFD1L, MTHFD2) in M1–MØ (Figure 7A). ABCC3 and ABCG2 show differential expression in M1–MØ over M2–MØ, whereas ABCC4 and ABCC5 are differentially expressed in M2–MØ over M1–MØ (Figure 7B). MTX exposure confers a marked increase in ABCB1/P-glycoprotein expression. This ABC transporter does not export MTX, but does extrude several inflammatory mediators (e.g., leukotrienes) [32]. 

Another series of pathways examined DGE in M1–MØ vs. M2–MØ and the effect of MTX therein is shown in Supplementary Figure S2 for (A) NFκB signaling pathway, (B) cytokines/chemokines, (C) signaling proteins, (D) immunometabolism-glycolysis, (E) pentose phosphate pathway, (F) TCA cycle and (G) membrane marker proteins. The effects of MTX were reflected in increased gene expression of TNFSF14, TNFAIP3, IL1RN, CCL22, CSF1, LIF, GDF15, INHBA, SOCS1, P2RX7, SUNCR1 and CD274 in M1–MØ. Energy metabolism in M1–MØ had a glycolysis-dependent profile. Membrane markers differentially expressed on M1–MØ included CD1B, CD52, IL2RG, and for M2–MØ: CD14, CD28, CD163, CD169 and CD209.

## 3. Discussion

The role of FPGS in MTX-polyglutamylation as a critical factor for the therapeutic efficacy of MTX in RA/JIA treatment is well established. However, little research has been performed on MTX metabolism in specific immune cell types involved in the pathophysiology of RA which may be targeted by MTX. In the present study, we specifically addressed FPGS pre-mRNA splicing in monocytes, M1- and M2-polarized macrophages. Deregulated splicing is being increasingly recognized as an emerging mechanism of drug resistance, including MTX, in oncology, but may also hold relevance for chronic inflammatory diseases [20,23]. To this end, increased expression of one particular FPGS splice variant (partial retention intron 8: 8PR) over FPGS WT had recently been associated with poor overall survival of pediatric acute lymphoblastic leukemia patients undergoing MTX-therapy [21]. In addition, increased 8PR/WT expression in PBMCs of RA patients was associated with higher disease activity following low-dose MTX therapy in RA patients [22]. Based on these results and those from the current study, it will be of interest to examine FPGS splicing and FPGS activity in monocyte subsets from RA patients at baseline and during MTX therapy to determine whether these parameters are associated with clinical response to MTX [37]. 

We observed a marked decrease in FPGS 8PR/WT expression during monocyte to macrophage differentiation and GM-CSF-skewed M1–MØ and M-CSF-skewed M2–MØ polarization. For macrophages, this decrease was inversely correlated with an increase in FPGS activity, being consistent with the notion that FPGS (8PR) splice variant translates into dysfunctional proteins [19]. Despite the fact that M1–MØ and M2–MØ harbor comparable FPGS enzyme activities, formation and accumulation of MTX-PGs was surprisingly much more prominent in M1–MØ than in M2–MØ; this may be explained by differential MTX uptake in these cells. In this respect, M1–MØ use RFC as the primary MTX influx transporter (Figure 7A) which is not affected by folic acid concentrations present in the cell culture medium, whereas M2–MØ express FOLR2/FRβ and PCFT as dominant MTX influx carriers, both of which are subject to a strong folic acid competition on uptake [9,27,38,39,40,41]. Moreover, the differential expression of ABC drug efflux transporters in M1–MØ and M2–MØ (Figure 7B) may have contributed to the extrusion of MTX that had not been converted to MTX-PG_2–3_ [42,43,44]. Collectively, these results demonstrate that additional factors other than FPGS splicing and FPGS activity may contribute to the accumulation of MTX-PGs in macrophages. 

Beyond specific FPGS splicing alterations, no investigations have been conducted regarding global mRNA splicing profiles for MTX-exposed polarized macrophages. RNA-seq analysis showed abundant splicing events in M1–MØ, M2–MØ and their MTX-exposed counterparts, which for MTX-exposed M2–MØ did not translate into large numbers of differentially expressed genes. Annotations of the splice events did not reveal specific folate metabolic pathways but rather cell cycle and Golgi vesicle transport (M1–MØ vs. M1–MØ + MTX) or histone modification and methylation (M2–MØ vs. M2–MØ + MTX), the latter of which exhibited higher confidence scores. These results indicate that although exposure to MTX did not cause substantial changes in gene expression profiles in M2–MØ, strong confidence can be seen in enrichment of differentially spliced genes involved in histone modification and/or methylation. As such, epigenetic mechanisms might underlie the disparity in gene expression after exposure to MTX between M1–MØ and M2–MØ [45]. 

DGE profiles (Supplemental Figure S2) confirmed previous studies on MTX-responsive genes (e.g., TNFAIP3, LIF, GDF15, and INHBA) in M1–MØ + MTX [6,25,46]. Similarly, GSEA analysis corroborated previous reports of MTX treatment inducing a more pro-inflammatory profile in M1–MØ + MTX [25,47]. This notion may be in accord with the mechanism of action of MTX by the non-lytic release of ATP and ADP. These phosphorylated adenine nucleotides elicit a pro-inflammatory response via activation of the purinergic system involving upregulation of G-protein-coupled receptors, e.g., P2X7R, which mediates NLRP3 inflammasome activation and cytokine release [4,48]. Extracellular ATP and ADP are eliminated by the action ecto-phosphatase CD39 on the cell surface which converts ATP and ADP into AMP and ultimately to adenosine via the activity of CD73, exerting an anti-inflammatory effect [4,49,50,51]. The markedly increased P2RX7 expression in M1–MØ + MTX is in support of this pro-inflammatory mechanism of MTX action (Supplemental Figure S2C). 

MTX also showed an impact on the expression of immunometabolism genes in pro-inflammatory M1–MØ that featured a higher glycolytic profile (Supplemental Figure S2D–F). M1–MØ are known for their ability to rewire their TCA cycle to produce regulatory immunometabolites [52,53,54]. Of particular interest was the effect of MTX on the succinate receptor SUCNR1/GPR91 (Supplemental Figure S2F). This receptor functions in sensing of extracellular succinate, a TCA cycle intermediate, extruded from pro-inflammatory macrophages [55,56]. Succinate serves as an inflammatory mediator for the NLRP3 inflammasome and induces IL-1β production [55,57]. A recent study showed that increasing plasma concentrations of the activated macrophage-derived TCA immunometabolite itaconate were associated with a poorer decrease in disease activity of RA patients following MTX treatment [58]. In addition, studies by Gosselt et al. [59] revealed higher plasma concentrations of the glycolytic intermediates (i.e., 1,3-/2,3-diphosphoglyceric acid, glycerol-3-phosphate and phosphoenolpyruvate) in RA patients with insufficient MTX response. These biochemical and genetic studies encourage further analysis of macrophage metabolic genes and immunometabolites as lead biomarkers for lack of response to MTX in RA patients. 

MTX predominantly targets folate metabolism which fuels multiple one-carbon donor reactions for fundamental processes including biosynthesis of purines and thymidylate (for DNA/RNA synthesis), amino acid biosynthesis (serine, glycine and methionine) and methylation reactions (S-adenosylmethionine, SAM) [9,60,61]. Folate/one carbon metabolism is compartmentalized in the cytosol and mitochondria [62], where intercompartmental communication preserves cytosolic folate homeostasis [63]. Both folate/1C and serine metabolism also support SAM-dependent histone methylation reactions and glutathione biosynthesis which promote IL-1β production in inflammatory macrophages [45,64]. Although MTX does not appear to enter mitochondria via MFT (SLC25A32), and thus does not undergo polyglutamylation by mitochondrial FPGS [65,66,67], MTX does affect mitochondrial folate metabolism by reducing the cytoplasmic folate pool of reduced folate cofactors [63]. Consistently, a crucial mitochondrial folate gene such as MTHFD2 showed an upregulated expression in inflammatory M1–MØ cells following MTX exposure (Figure 7A). Together, exposure of M1–MØ to low-dose MTX has impact on folate pathway and other downstream metabolic and signalling pathways which apparently contribute to a recently described macrophage reprogramming ability of MTX; the latter confers a state of LPS tolerance associated with higher soluble CD14 levels in the plasma of MTX responding RA patients [25]. 

Finally, this study has several limitations. First, monocytes used for skewing to polarized macrophages were derived from healthy individuals rather than from therapy-naïve or (short/long term) MTX-treated RA patients. Second, one type of stimulus was used for monocyte-macrophages (GM-CSF and M-CSF) where alternative stimuli (e.g., IFNγ, IL-10, LPS, or synovial fluid) also warrant further exploration. Third, all readouts for polarized macrophages were done for one fixed time point of 7 days incubation with one low-dose of MTX (50 nmol/L) and cells maintained in cell culture medium containing supraphysiological concentrations of folic acid rather than a defined culture medium supplemented with a physiological reduced folate cofactor at concentrations mimicking the 10–30 nmol/L plasma levels of 5-methyl-THF [68]. Designing experimental conditions taking into account these considerations may reveal the dynamics of how MTX controls folate and other pathways in various immune cells and provide an optimal therapeutic effect for RA. 

**Figure 8:**
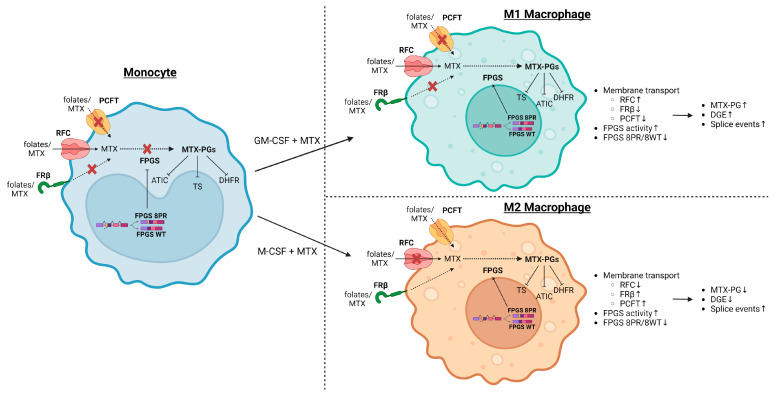
Schematic summary model of MTX-induced alterations in folate metabolism in M1–MØ and M2–MØ with emphasis on the role of FPGS splicing and enzyme activity as well as folate transporters. MTX is taken up by monocytic/macrophage cells by either of 3 transporters; Reduced Folate Carrier (RFC), primarily expressed on M1–MØ, and proton-coupled folate transporter (PCFT) and Folate Receptor β (FRβ), primarily expressed on M2–MØ. Following uptake, MTX is polyglutamylated by FPGS, of which the activity is low in monocytes due to increased FPGS splicing (8PR splice variant). During monocyte–macrophage differentiation, the ratio of 8PR/WT FPGS splicing decreases, resulting in higher FPGS activity conferring MTX-PG formation inhibiting key target enzymes (TS, DHFR, ATIC) in folate metabolism. Image created via BioRender.

Altogether, a model depicted in Figure 8 summarizes the MTX-polyglutamylation process in M1 and M2 macrophages in light of the role of FPGS splicing and enzyme activity, and folate transporters. 

## 4. Materials and Methods

### 4.1. Materials

Reagents were obtained from the following sources: Methotrexate (Emthexate PF, Teva B.V., Haarlem, The Netherlands), M-CSF and GM-CSF (ImmunoTools, Friesoythe, Germany), ^15^N-labeled L-glutamic acid (Sigma-Aldrich, St. Louis, MO, USA), Lymphoprep (Nycomed Pharma, Oslo, Norway). 

### 4.2. Monocyte-Macrophage Polarization Studies

Human peripheral blood mononuclear cells (PBMCs) were isolated from buffy coats from healthy individuals in accordance with national government guidelines on human research. PBMCs were isolated from buffy coats using Lymphoprep (Nycomed Pharma, Oslo, Norway) density gradient centrifugation and then collected in standard RPMI-1640 medium (with 1 mg/mL folic acid, Gibco, Life Technologies Ltd., Paisley, UK) supplemented with 10% fetal calf serum (FCS) (Greiner Bio-One, Alphen aan den Rijn, The Netherlands), 2 mmol/L L-glutamine (Gibco, Life Technologies Corporation, Grand Island, NE, USA). 

Procedures for monocyte isolation and macrophage skewing were carried out essentially as described previously [6,46,69]. Briefly, monocytes were purified from PBMCs by magnetic cell sorting using CD14 microbeads and were cultured at 5 × 10^5^ cells/mL for 7 days in standard RPMI-1640 medium (with 2.2 μmol/L folic acid) Supplemented with 10% fetal calf serum, and containing GM-CSF (1000 U/mL) to generate GM-CSF-polarized macrophages (GM–MØ, M1–MØ) or M-CSF (10 ng/mL) to generate M-CSF-polarized macrophages (M–MØ, M2–MØ). GM-CSF or M-CSF was added every two days. These cultures were incubated without or with low-dose (50 nmol/L) or high-dose (5 μmol/L) MTX and harvested at day 7 for various analyses. 

### 4.3. RNA Isolation and RNA-Seq

RNA was isolated from monocytes and GM–MØ/M1 and M–MØ/M2 macrophages (with or without 7 days exposure to 50 nmol/L MTX) from 3 individual donors using the RNeasy Mini Kit (Qiagen, Hilden, Germany).

Libraries for RNA-seq were generated with the SMARTer Stranded Total RNA-Seq Kit–Pico Input Mammalian (Takara Bio USA, Mountain View, CA, USA) following the manufacturer’s protocol. Libraries were then pooled and sequenced on a HiSeq 4000 sequencing system (Illumina, San Diego, CA, USA) with a 150-base pair paired-end (2 × 150 PE) protocol. Resulting FASTQ files were trimmed using trimmomatic [70]. 

### 4.4. RNA-Seq Analysis

Alignment of FASTQ files was performed using the default settings of STAR (Version 2.4) [71], except the following options: –alignEndsType EndToEnd to remove soft-clipping of the reads, -outSAMtype BAM to produce .bam files and –sdjbOverhang 150 for optimal splice junction overhang length. Human genome version hg38 was used as reference genome [72]. Differential gene expression (DGE) was calculated with R package edgeR (Version 3.13) [73] using linear model fits for gene expression comparisons. Log fold changes (logFC) larger than 1.5 and *p*-values smaller than 0.05 were considered significant events. rMATS (Version 4.0.2) [74,75,76] was used to perform alternative splicing analysis between groups with default settings. This software detects sequencing reads and classifies them into 4 distinct splice event categories: skipped exons (SE), retained introns (RI), alternative 3′ splice site (A3SS) and alternative 5′ splice site (A5SS). Inclusion Level Differences > 0.1 and FDR < 0.01 were considered statistically significant. Gene ontology terms for potential protein networks using differentially spliced genes was performed in String [77]. For gene set enrichment analysis (GSEA), gene sets available on the website were used [78]. The data discussed in the current paper have been deposited in NCBI’s Gene Expression Omnibus [79] and are accessible through GEO Series accession number GSE188278 and GSE228475.

### 4.5. qPCR Analysis

Isolated RNA was reverse transcribed to cDNA with the High Capacity cDNA Reverse Transcription Kit (Applied Biosystems, Foster City, USA). Pre-mRNA expression of FPGS 8PR/8WT was examined by quantitative polymerase chain reaction (qPCR) analysis as described by Muller et al. [22]. In short, 20 µL qPCR mixtures containing 12.5 ng cDNA, 10 µL LightCycler 480 SYBR Green I Master Mix (2× concentrated, Roche Diagnostics GmbH, Mannheim, Germany), 1 µL of 5 µmol/L forward primer (Biolegio B.V., Nijmegen, The Netherlands), 1 µL of 5 µmol/L reverse primer (Biolegio B.V., Nijmegen, The Netherlands) and 3 µL RNase-free ddH_2_O were run on a LightCycler^®^ 480 (Roche Diagnostics GmbH, Mannheim, Germany). Β-glucuronidase (GUSB) was used as reference gene. Relative gene expression analyses were performed on the LightCycler^®^ 480 software (version 1.5.1.62, Roche) with the Advanced Relative Quantification module.

### 4.6. FPGS Catalytic Assay

FPGS enzymatic activity in monocytes and macrophages was determined via an LC-MS/MS based analysis assay as described in detail by Muller et al., [16]. In short, the assay mixture consists of cellular extracts of 0.8–2 × 10^6^ monocytes/macrophages (10–20 μg protein/assay) and FPGS substrates ^15^N-L-glutamic acid (Sigma-Aldrich, St. Louis, USA, final concentration: 4 mmol/L) and MTX (Emthexate PF, Teva B.V., Haarlem, The Netherlands, final concentration: 250 μmol/L) incubated at pH 8.85 and 37 °C for 2 hrs. The FPGS reaction product ^15^N-MTX-PG_2_ was quantified by LC-MS/MS and FPGS activity and was expressed as pmol ^15^N-MTX-PG_2_ formed/h/mg protein. FPGS activity in the human leukemia cell line CCRF-CEM and its FPGS-deficient subline CEM/R30dm served as positive and negative reference [16]. 

### 4.7. LC-MS/MS MTX-PG Analysis

MTX-PG formation in GM–MØ/M1 and M–MØ/M2 macrophages incubated for 7 days with low-dose (50 nmol/L) or high-dose (5 μmol/L) MTX was measured by LC-MS/MS methodologies as described in detail by Driehuis et al. [80] and Hebing et al. [81]. Briefly, frozen pellets of 0.8–2 × 10^6^ macrophages were thawed and resuspended in 25 μL ice-cold ddH_2_O, after which 25 μL of 25 nmol/L ^13^C_5_^15^N-labeled custom-made stable isotopes of MTX-PG_1–6_ were added as internal standards. After protein precipitation with 40 μL 16% perchloric acid on ice and centrifugation, the supernatant was assayed for individual MTX-PGs by LC-MS/MS. Quantification of the intracellular MTX-PG_1-6_ concentrations were expressed per cell number. 

### 4.8. Statistics

One-way ANOVA was used to identify significant differences between monocyte and macrophage fractions and MTX treatments. To determine statistical differences between each comparison, Tukey’s test was performed as post hoc analysis. An adjusted *p*-value < 0.05 was considered significant (*, *p* < 0.05; **, *p* < 0.01, ***, *p* < 0.001). Statistical analysis was performed with R Version 4.0.5 or GraphPad Prism Version 9.3.1 (GraphPad Software Inc., La Jolla, USA). 

## Data Availability

The data set supporting the conclusions of this article is available in the Gene Expression Omnibus repository (http://www.ncbi.nlm.nih.gov/geo/ accessed on 29 March 2023) under accession number GSE188278 and GSE228475.

## Supplementary Materials

The supporting information can be downloaded at: https://www.mdpi.com/article/10.3390/ijms24119641/s1.
