# Supplementary material for: Methotrexate Provokes Disparate Folate Metabolism Gene Expression and Alternative Splicing in Ex Vivo Monocytes and GM-CSF- and M-CSF-Polarized Macrophages
Source: Int J Mol Sci. 2023 Jun 1;24(11):9641. doi: 10.3390/ijms24119641 (PMC10253671; doi:10.3390/ijms24119641)
Supplement: Supplementary file 1 [file ijms-24-09641-s001.zip › IJMS-2374281R1 Table Suppl Table S8 GSEA report M1_M1MTX.pdf]

	GS<br>follow link to MSigDB	SIZE	ES	NES	NOM<br>p-val	FDR<br>q-val	FWER<br>p-val	RANK AT<br>MAX	LEADING EDGE
1	HALLMARK_TNFA_SIGNALING_VIA_NFKB	185	-0.71	-2.67	0.000	0.000	0.000	2072	tags=54%, list=14%, signal=61%
2	HALLMARK_INFLAMMATORY_RESPONSE	165	-0.61	-2.29	0.000	0.000	0.000	2280	tags=41%, list=15%, signal=48%
3	HALLMARK_P53_PATHWAY	183	-0.61	-2.29	0.000	0.000	0.000	2289	tags=38%, list=15%, signal=44%
4	HALLMARK_EPITHELIAL_MESENCHYMAL_TRANSITION	127	-0.59	-2.12	0.000	0.000	0.000	2233	tags=42%, list=15%, signal=48%
5	HALLMARK_INTERFERON_ALPHA_RESPONSE	93	-0.60	-2.06	0.000	0.000	0.000	3368	tags=51%, list=22%, signal=64%
6	HALLMARK_INTERFERON_GAMMA_RESPONSE	183	-0.50	-1.89	0.000	0.000	0.001	2846	tags=40%, list=19%, signal=48%
7	HALLMARK_ESTROGEN_RESPONSE_LATE	142	-0.47	-1.69	0.000	0.006	0.033	2445	tags=29%, list=16%, signal=34%
8	HALLMARK_COAGULATION	90	-0.49	-1.66	0.001	0.008	0.055	2230	tags=38%, list=15%, signal=44%
9	HALLMARK_HYPOXIA	158	-0.45	-1.66	0.000	0.008	0.058	2301	tags=28%, list=15%, signal=32%
10	HALLMARK_APOPTOSIS	141	-0.46	-1.66	0.001	0.008	0.063	2175	tags=26%, list=14%, signal=30%
11	HALLMARK_UV_RESPONSE_UP	133	-0.45	-1.62	0.001	0.010	0.092	3925	tags=40%, list=26%, signal=53%
12	HALLMARK_COMPLEMENT	163	-0.43	-1.60	0.000	0.012	0.114	1894	tags=21%, list=12%, signal=24%
13	HALLMARK_IL6_JAK_STAT3_SIGNALING	70	-0.48	-1.58	0.005	0.013	0.139	1707	tags=26%, list=11%, signal=29%
14	HALLMARK_ALLOGRAFT_REJECTION	149	-0.43	-1.58	0.001	0.013	0.148	2467	tags=26%, list=16%, signal=31%
15	HALLMARK_KRAS_SIGNALING_DN	80	-0.46	-1.56	0.006	0.015	0.179	1656	tags=20%, list=11%, signal=22%
16	HALLMARK_IL2_STAT5_SIGNALING	167	-0.42	-1.55	0.004	0.015	0.193	2537	tags=28%, list=17%, signal=33%
17	HALLMARK_UNFOLDED_PROTEIN_RESPONSE	107	-0.44	-1.55	0.004	0.016	0.216	4432	tags=40%, list=29%, signal=56%
18	HALLMARK_KRAS_SIGNALING_UP	140	-0.43	-1.55	0.003	0.015	0.218	2165	tags=25%, list=14%, signal=29%

19	HALLMARK_ESTROGEN_RESPONSE_EARLY	150	-0.40	-1.45	0.009	0.040	0.480	1857	tags=19%, list=12%, signal=22%
20	HALLMARK_UV_RESPONSE_DN	115	-0.41	-1.45	0.016	0.039	0.490	2069	tags=20%, list=14%, signal=23%
21	HALLMARK_APICAL_JUNCTION	133	-0.39	-1.41	0.018	0.055	0.641	902	tags=14%, list=6%, signal=14%
22	HALLMARK_MYOGENESIS	123	-0.39	-1.40	0.024	0.058	0.680	2973	tags=27%, list=19%, signal=33%
23	HALLMARK_ANDROGEN_RESPONSE	87	-0.40	-1.33	0.077	0.097	0.864	2590	tags=23%, list=17%, signal=28%
24	HALLMARK_MTORC1_SIGNALING	196	-0.35	-1.32	0.035	0.102	0.889	4203	tags=34%, list=27%, signal=46%
25	HALLMARK_MYC_TARGETS_V2	56	-0.40	-1.28	0.123	0.137	0.955	5118	tags=61%, list=33%, signal=91%
26	HALLMARK_XENOBIOTIC_METABOLISM	144	-0.35	-1.26	0.076	0.151	0.971	4013	tags=35%, list=26%, signal=48%
27	HALLMARK_CHOLESTEROL_HOMEOSTASIS	67	-0.38	-1.24	0.146	0.172	0.984	2972	tags=22%, list=19%, signal=28%
28	HALLMARK_WNT_BETA_CATENIN_SIGNALING	34	-0.37	-1.07	0.349	0.485	1.000	2479	tags=26%, list=16%, signal=32%
29	HALLMARK_DNA_REPAIR	144	-0.29	-1.04	0.381	0.541	1.000	4121	tags=31%, list=27%, signal=42%
30	HALLMARK_SPERMATOGENESIS	74	-0.31	-1.04	0.406	0.531	1.000	1529	tags=14%, list=10%, signal=15%
31	HALLMARK_HEDGEHOG_SIGNALING	24	-0.39	-1.02	0.443	0.564	1.000	692	tags=13%, list=5%, signal=13%
32	HALLMARK_TGF_BETA_SIGNALING	49	-0.31	-0.97	0.496	0.666	1.000	2514	tags=16%, list=16%, signal=19%
33	HALLMARK_PI3K_AKT_MTOR_SIGNALING	89	-0.27	-0.92	0.599	0.785	1.000	3388	tags=20%, list=22%, signal=26%
34	HALLMARK_ANGIOGENESIS	22	-0.34	-0.90	0.630	0.817	1.000	2594	tags=32%, list=17%, signal=38%
35	HALLMARK_GLYCOLYSIS	162	-0.23	-0.86	0.752	0.866	1.000	1706	tags=10%, list=11%, signal=12%
36	HALLMARK_PEROXISOME	86	-0.25	-0.85	0.756	0.878	1.000	2547	tags=17%, list=17%, signal=21%
37	HALLMARK_ADIPOGENESIS	178	-0.23	-0.84	0.825	0.865	1.000	1412	tags=8%, list=9%, signal=9%

38	HALLMARK_NOTCH_SIGNALING	29	-0.26	-0.73	0.863	1.000	1.000	1603	tags=14%, list=10%, signal=15%
39	HALLMARK_FATTY_ACID_METABOLISM	132	-0.18	-0.64	0.995	1.000	1.000	1821	tags=8%, list=12%, signal=9%
40	HALLMARK_MYC_TARGETS_V1	199	-0.15	-0.58	1.000	1.000	1.000	5948	tags=40%, list=39%, signal=65%
41	HALLMARK_PROTEIN_SECRETION	90	-0.13	-0.46	1.000	1.000	1.000	4815	tags=27%, list=31%, signal=39%
