# Supplementary material for: Methotrexate Provokes Disparate Folate Metabolism Gene Expression and Alternative Splicing in Ex Vivo Monocytes and GM-CSF- and M-CSF-Polarized Macrophages
Source: Int J Mol Sci. 2023 Jun 1;24(11):9641. doi: 10.3390/ijms24119641 (PMC10253671; doi:10.3390/ijms24119641)
Supplement: Supplementary file 1 [file ijms-24-09641-s001.zip › ijms-2374281-supplementary figures S1-S2.pdf]

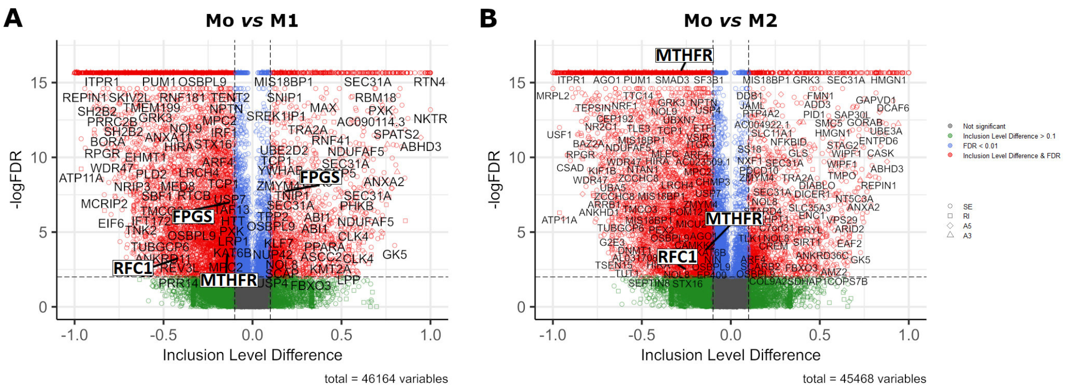

Figure S1. Volcano plots of differentially spliced events (SE, RI, A3 and A5) for (**A**) Mo *vs* M1-MØ, and (**B**) Mo *vs* M2-MØ. Results depicted are the means of 3 individual donors.

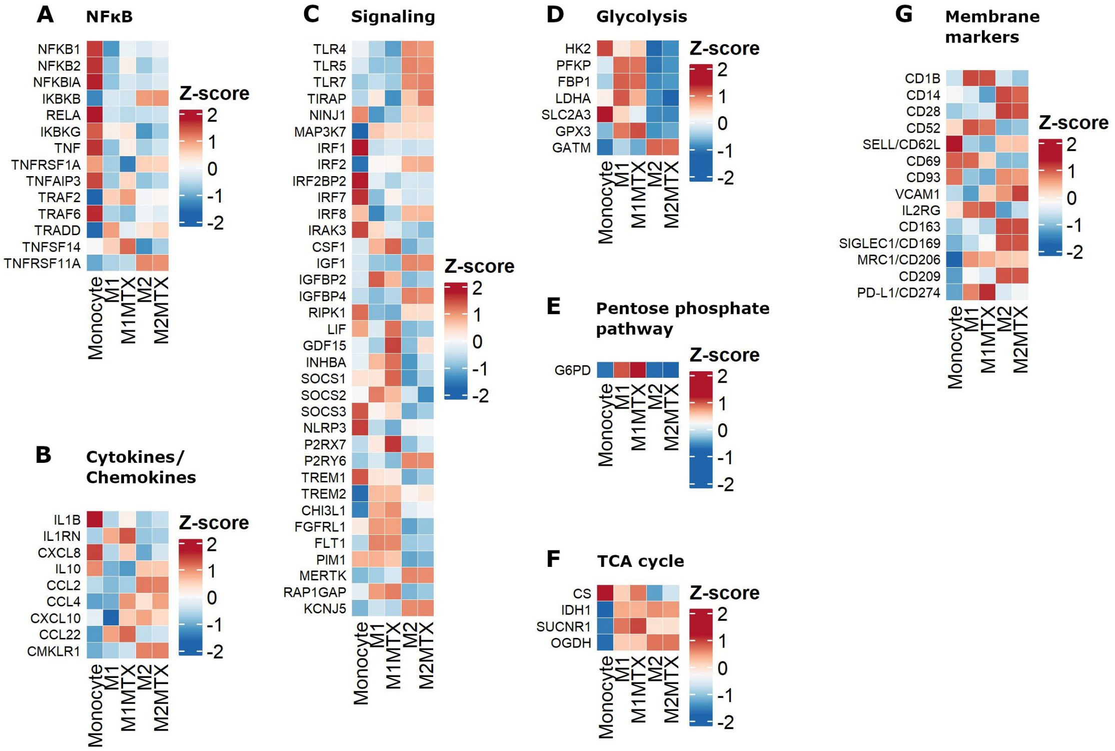

Figure S2. Heat maps of differential gene expression of Mo, M1-MØ, M1-MØ + MTX, M2-MØ and M2-MØ + MTX for (**A**) genes involved in NFκB signaling pathway, (**B**) cytokines/chemokines, (**C**) signaling pathway proteins, (**D**) immunometabolism: glycolysis, (**E**) pentose phosphate pathway, (**F**) TCA cycle, and (**G**) Cluster of Differentiation (CD) membrane marker proteins. Results depicted are the means of 3 individual donors.

Table S1: Top 100 differentially spliced events (SE, RI, A3 and A5; Inclusion Level Difference < 0.1) for Mo *vs* M1-MØ.

Table S2: Top 100 differentially spliced events (SE, RI, A3 and A5; Inclusion Level Difference < 0.1) for Mo *vs* M2-MØ.

Table S3: Top 100 differentially spliced events (SE, RI, A3 and A5; Inclusion Level Difference < 0.1) for M1-MØ *vs* M2-MØ.

Table S4: Top 100 differentially spliced events (SE, RI, A3 and A5; Inclusion Level Difference < 0.1) for M1-MØ *vs* M1-MØ + MTX.

Table S5: Top 100 differentially spliced events (SE, RI, A3 and A5; Inclusion Level Difference < 0.1) for M2-MØ *vs* M2-MØ + MTX.

Table S6: Top 100 differentially spliced events (SE, RI, A3 and A5; Inclusion Level Difference < 0.1) for M1-MØ + MTX *vs* M2-MØ + MTX.

Table S7: Differential gene expression top 100 genes up- or down-regulated (FDR < 0.05) in Mo, M1-MØ, M1-MØ + MTX, M2-MØ and M2-MØ + MTX.

Table S8: GSEA report for pre-ranked list (FDR < 0.05) of all DEG events of M1-MØ *vs* M1-MØ + MTX.
